# A new light on the UFO mystery: *Zmufo1* encodes a nuclear protein that modulates redox levels and epigenetic status during basal endosperm differentiation in maize

**DOI:** 10.1093/plcell/koae307

**Published:** 2024-11-19

**Authors:** Nicolas M Doll

**Affiliations:** The Plant Cell, American Society of Plant Biologists; Laboratoire Reproduction et Développement des Plantes, Univ Lyon, ENS de Lyon, UCB Lyon 1, CNRS, INRAE, F-69342, Lyon, France

Maize is the most widely produced cereal globally (https://www.fao.org/faostat/), largely due to the high starch and protein content stored in its kernels, primarily within the endosperm. Nutrient loading into the endosperm takes place in a specialized tissue known as the basal endosperm transfer layer (BETL). BETL cells develop numerous cell wall ingrowths that increase the surface area for nutrient exchange with the adjacent maternal tissue and express transporters essential for nutrient import during endosperm filling. Consequently, impaired BETL differentiation leads to a drastic reduction in endosperm filling (Dai et al. 2021).

One of the key genes involved in BETL differentiation is *Unstable factor for orange 1* (*Zmufo1*). *Zmufo1* is strongly expressed in the BETL during differentiation, and its loss-of-function mutant, *ufo1-Dsg*, disrupts BETL differentiation (Chatterjee et al. 2021). *Zmufo1* was originally identified from the *Ufo1-1* mutant allele, which exhibits ectopic expression of *Zmufo1* in various tissues of the plant, due to the insertion of a transposable element in its first intron (Wittmeyer et al. 2018). Mis-expression of *Zmufo1* leads to growth defects, stress-related phenotypes, and hyperaccumulation of red-orange phlobaphene pigments, with variable penetrance. Notably, *Ufo1-1* kernels can be darkly pigmented, as *Zmufo1* misexpression in the pericarp activates the expression of *pericarp color 1* (*Zmp1*), the master regulator of phlobaphene biosynthesis (Chopra et al. 2003).

How *Zmufo1* regulates BETL differentiation and thereby contributes to grain filling has remained poorly understood. To address this, Debamalya Chatterjee and colleagues analyzed the effect of *Zmufo1* loss-of-function and overexpression on BETL differentiation using the *ufo1-Dsg* and *Ufo1-1* lines, respectively (Chatterjee et al. 2024). Both mutant lines exhibited impaired BETL cell differentiation but showed distinct defects in cell wall ingrowths. The *Ufo1-1* overexpressor line displayed excessively high expression of several BETL marker genes, while the loss-of-function *ufo1-Dsg* showed reduced or mis-expression of BETL marker genes, indicating a positive role of *Zmufo1* in BETL identity. In both *ufo1-Dsg* and *Ufo1-1* lines, the basal region of the kernel exhibits abnormally high levels of DNA damage and reactive oxygen species. Transcriptomic analyses revealed the misregulation of several genes involved in redox homeostasis, indicating an imbalance in redox potential when *Zmufo1* levels diverged from normal. Interestingly, treatments with antioxidant molecules glutathione and ascorbic acid strongly alleviated growth defects and DNA damage in both lines, indicating that *Zmufo1* regulates BETL differentiation and prevents DNA damage by controlling redox homeostasis.

The authors next aimed to identify the mode of action of *Zmufo1*. In the basal endosperm of both *Ufo1-1* and *ufo1-Dsg*, increased histone acetyltransferase activity was detected, suggesting a role of *Zmufo1* in epigenetic regulation. Consistent with this hypothesis, transmission electron microscopy revealed an altered heterochromatin pattern in the mutant lines compared with the wild type. The authors then analyzed the epigenetic marks on *Zmp1*, a known target of *Zmufo1*, in the pericarp of *Ufo1-1*. They observed reduced levels of histone 3 methylation in the promoter region, UTR, and specific introns of *Zmp1* compared with wild type, confirming the link between *Zmufo1* and epigenetic regulation.

Finally, the authors investigated the nature of the protein encoded by *Zmufo1*. A substantial part of the ZmUFO1 protein consists of a predicted intrinsically disordered region. Interestingly, at temperatures below 21 °C, in vitro–produced ZmUFO1 undergoes liquid-liquid phase separation and forms aggregates. Circular dichroism analysis on these aggregates revealed that ZmUFO1 contained approximately 38% of β-sheets, suggesting that a unique conformational structure underlies this phase separation at low temperatures. In plant cells, ZmUFO1 localizes in the nucleus, specifically along the nucleolus rim, and interacts with numerous nuclear proteins, including histone acetyl-transferases involved in epigenetic regulation and many proteins containing redox-sensitive cysteines.

In summary, Chatterjee and colleagues found that *Zmufo1* encodes a nuclear protein that regulates aspects of the cell's epigenetic status and redox potential, likely through physical interactions with other nuclear proteins involved in these processes. Through this regulation, *zmufo1* positively influences the expression of BETL genes and the differentiation of this crucial tissue for kernel filling (Fig.). One key point raised by this article is the temperature-sensitive ability of ZmUFO1 proteins to undergo phase separation. Further studies should be conducted to determine whether this phenomenon also occurs in vivo, and, if so, to understand its impact on the structure, activity, and effects of ZmUFO1 on BETL differentiation at different temperatures.

**Figure.**
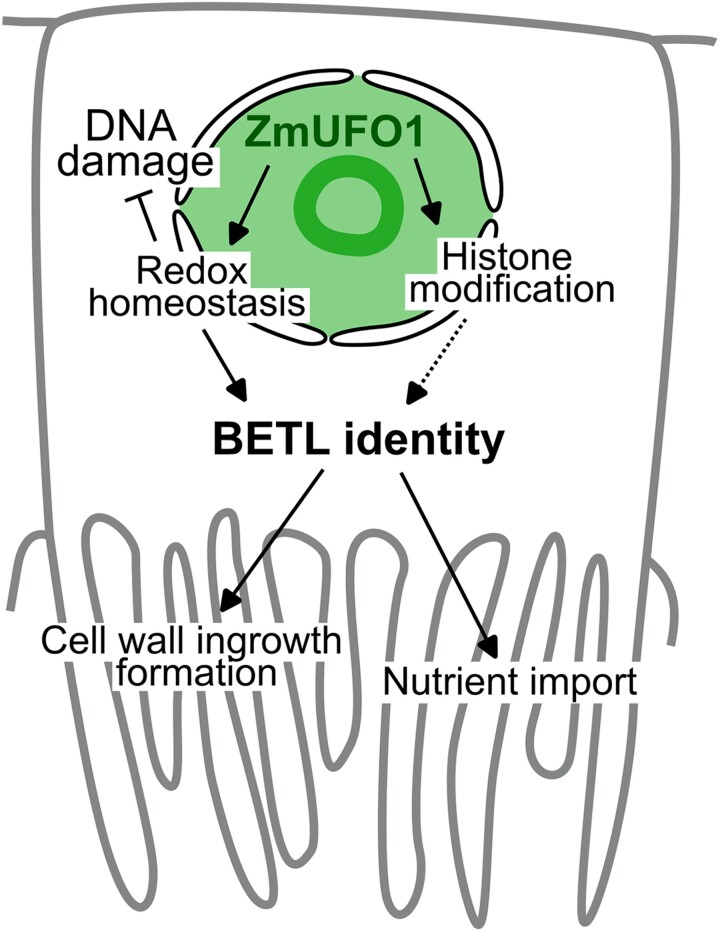
Role of *zmufo1* in BETL cell differentiation. *Zmufo1* regulates the cellular redox homeostasis and epigenetic landscape to promote BETL differentiation and prevent DNA damage. The colored area represents the localization of the ZmUFO1 protein. Figure credit: N. Doll.

## Data Availability

N/A.

## References

[koae307-B1] Chatterjee D , Wittmeyer K, Lee T-F, Cui J, Yennawar NH, Yennawar HP, Meyers BC, Chopra S. Maize unstable factor for orange1 is essential for endosperm development and carbohydrate accumulation. Plant Physiol. 2021:186(4):1932–1950. 10.1093/plphys/kiab183 33905500 PMC8331166

[koae307-B2] Chatterjee D , Zhang Z, Lin PY, Wang PH, Sidhu GK, Yennawar NH, Hsieh J-WA, Chen P-Y, Song R, Meyers BC, et al Maize *unstable factor for orange1* encodes a nuclear protein that affects redox accumulation during kernel development. Plant Cell. 2024: In press. 10.1093/plcell/koae301 PMC11663571 39589935

[koae307-B3] Chopra S , Cocciolone SM, Bushman S, Sangar V, McMullen MD, Peterson T. The maize unstable factor for orange1 is a dominant epigenetic modifier of a tissue specifically silent allele of pericarp color1. Genetics. 2003:163(3):1135–1146. 10.1093/genetics/163.3.1135 12663550 PMC1462483

[koae307-B4] Dai D , Ma Z, Song R. Maize endosperm development. J Integr Plant Biol. 2021:63(4):613–627. 10.1111/jipb.13069 33448626

[koae307-B5] Wittmeyer K , Cui J, Chatterjee D, Lee TF, Tan Q, Xue W, Jiao Y, Wang PH, Gaffoor I, Ware D, et al The dominant and poorly penetrant phenotypes of maize unstable factor for orange1 are caused by DNA methylation changes at a linked transposon. Plant Cell. 2018:30(12):3006–3023. 10.1105/tpc.18.00546 30563848 PMC6354275

